# Supplementary material for: Pathway Polygenic Risk Scores (pPRS) for the Analysis of Gene-environment Interaction
Source: bioRxiv. 2024 Dec 19:2024.12.16.628610. Preprint. [Version 1] doi: 10.1101/2024.12.16.628610 (PMC11702571; doi:10.1101/2024.12.16.628610)
Supplement: Supplement 1 [file NIHPP2024.12.16.628610v1-supplement-1.pdf]

bioRxiv preprint doi: https://doi.org/10.1101/2024.12.16.628610; this version posted December 20, 2024. The copyright holder for this preprint (which was not certified by peer review) is the author/funder, who has granted bioRxiv a license to display the preprint in perpetuity. It is made available under aCC-BY 4.0 International license.

Table S1: Estimated type I error and interaction effect for PRSxE using different PRS construction methods

PRS SNPs	Type I Error* PRS x E	Estimated $\beta_{PRS\times E}$
GWAS significant SNPs under threshold:		
5×10^{-5}	0.054	-0.0036
5×10^{-3}	0.038	-0.0049
5×10^{-1}	0.056	-0.0033
20 main effects SNPs	0.050	-0.0029
10 main effects SNPs and 10 no-effects SNPs	0.050	0.0033

* Estimated Type I error and interaction effect based on 1,000 simulated replicates. Each replicate includes 20 SNPs with only main effects and no interaction
